# Construction and validation of nomogram to predict distant metastasis in osteosarcoma: a retrospective study

**DOI:** 10.1186/s13018-021-02376-8

**Published:** 2021-03-30

**Authors:** Shouliang Lu, Yanhua Wang, Guangfei Liu, Lu Wang, Pengfei Wu, Yong Li, Cai Cheng

**Affiliations:** 1 NO.1 Orthopedics Department, Cangzhou Central Hospital, Cangzhou, Hebei Province China; 2 ECG Examination Department, Cangzhou Central Hospital, Cangzhou, Hebei Province China

**Keywords:** Osteosarcoma, Metastasis, Logistic regression, Nomogram

## Abstract

**Background:**

Osteosarcoma is most common malignant bone tumors. OS patients with metastasis have a poor prognosis. There are few tools to assess metastasis; we want to establish a nomogram to evaluate metastasis of osteosarcoma.

**Methods:**

Data from the Surveillance, Epidemiology, and End Results (SEER) database of patients with osteosarcoma were retrieved for retrospective analysis. We identify risk factors through univariate logistic regression and multivariate logistic regression analysis. Based on the results of multivariate analysis, we established a nomogram to predict metastasis of patients with osteosarcoma and used the concordance index (C-index) and calibration curves to test models.

**Results:**

One thousand fifteen cases were obtained from the SEER database. In the univariate and multivariate logistic regression analysis, age, primary site, grade, T stage, and surgery are risk factors. The nomogram for metastasis was constructed based on these factors. The C-index of the training and validation cohort was 0.754 and 0.716. This means that the nomogram predictions of patients with metastasis are correct, and the calibration plots also show the good prediction performance of the nomogram.

**Conclusion:**

We successfully develop the nomogram which can reliably predict metastasis in different patients with osteosarcoma and it only required basic information of patients. The nomogram that we developed can help clinicians better predict the metastasis with OS and determine postoperative treatment strategies.

## Introduction

Osteosarcoma (OS) originates from skeleton system throughout the body, especially in children and adolescents during bone growth [1] and a second incidence peak after 50 years [2]. OS is always the most common primary malignant tumor pathology of the skeleton system. It is generally believed that metastasis is an important factor affecting the prognosis of osteosarcoma patients [3]. Since the chemotherapy was applied to cancer therapy, the prognosis of non-metastatic osteosarcoma patients was obviously improved [4]. However, the osteosarcoma with metastasis is still poor [3, 5]. For instance, comparing to the 70% 5-year overall survival of non-metastasis osteosarcoma, the OS patients with lung metastasis is only 30% [6].

The treatment strategy has some difference between metastatic and non-metastatic OS patients. Surgical treatment has always been the standard treatment for osteosarcoma [7]. However, when the tumor recurs locally or metastases to the lungs and cannot be removed, radiotherapy or chemotherapy can be considered firstly [8]. However, there is no useful method to evaluate metastasis status. Therefore, it is urgent to develop tools to predict the distant metastasis of osteosarcoma to guide clinical work.

The nomogram is a statistical tool; it can combine all independent risk factors to evaluate the endpoint accident in which we are interested. Nowadays, nomograms have been widely applied to predict the metastasis of other cancer patients such as renal cell carcinoma [9], gastrointestinal stromal tumor [10], and thyroid carcinoma [11].

## Materials and methods

### Data source and inclusion criteria

Demographic and clinicopathological characteristics of osteosarcoma patients were obtained from Surveillance, Epidemiology, and End Results (SEER) database. The data on cancer patients that is freely available in the SEER database comes from cancer registries in 18 regions, which account for approximately 30% of the US population. The database includes patients’ demographic characteristics, tumor pathological characteristics, therapy details, and follow-up records [12]. We finally selected 1015 osteosarcoma cases from the SEER database according to the following included criteria: (a) all patients were diagnosed between 2010 and 2015, (b) all patients were diagnosed with primary osteosarcoma by pathology or clinical, (c) the metastasis status is clear, and (d) completed follow-up. Exclusion criteria were as follows: (a) pathology is not osteosarcoma and (b) unknown age, race, sex, tumor size, primary site, grade, and T stage.

### Study variables

The variables we included in the study are age at diagnosis, sex, race, tumor size (CS tumor size, 2004+), primary site, metastasis, T stage, and surgery. Age at diagnosis was divided into under 20 years old, 20–49 years old, and over 50 years old. Race classification is white, black, and other. The pathological grade was divided into high grade (including grades I–II) and low grade (including grades III–IV) according to the variable “ICD-O-3 grade”. The tumor size is classified to <5cm, 5–10cm, and ≥10cm in terms of the variable. The primary site was classified into external, axial, and other. The T stage includes T1, T2, and T3 according to Derived AJCC T, 7th ed. Surgery means the surgery information of primary site.

### Statistical analysis

All patients (*n* = 1015) were randomly divided into training cohort (*n* = 610) and validation cohort (*n* = 405) to construct and validate the nomogram, separately. Kaplan–Meier survival analysis was performed between metastasis and non-metastasis patients. Metastasis means that osteosarcoma metastasizes to distant site. We used univariate logistic regression analysis and log rank test to identify potential factors that impact on metastasis of patients. The meaningful risk factors which were selected from logistic regression analysis were further analyzed by the multivariate logistic regression analysis to confirm independent risk factors. The logistic regression model is used to calculate the hazard ratio of each variable with a corresponding 95% confidence interval (CI). To estimate the effect of multicollinearity, we calculate the kappa coefficient of the model in train cohort and the value is 8.081531 which represents multicollinearity is weak. We apply the stepwise regression to select the best logistic regression model. Randomized grouping and univariate and multivariate regression were performed using R version 4.0.2 (https://www.r-project.org/).

### Development and validation of nomogram

Based on the results obtained from the multivariate logistic regression analysis, we constructed the nomogram to predict the metastasis risk. This study constructed the nomogram through the training cohort and then validated it through the validation queue to test its accuracy. The index of concordance (C-index) which reflects on the possibility of consistency between predicted probability and observed outcome can be used to evaluate the predictive performance of nomogram. The C-index value ranges from 0.5 to 1.0, where 0.5 represents random and 1.0 represents a perfect match. The higher the C-index value, the higher the consistency between the prediction and the observed result. The C-index is at least 0.7 and the nomogram prediction is meaningful. At the same time, internal calibration plot and external validation cohort are also used to evaluate the predictive ability of nomogram. The nomogram, receiver operating characteristic (ROC) curve, and calibration curve were performed using R version 4.0.2 (https://www.r-project.org/). A two-sided *P*<0.05 was considered statistically significant.

## Results

### Patient baseline characteristics

We finally selected 1015 eligible osteosarcoma cases from the SEER database according to the included criteria. These data were randomly divided into a training cohort (*n* = 610) and a validation cohort (*n* = 405). As show in Table 1, 484 (47.68%) patients were aged < 20, 327 (32.22%) patients are between 20 and 49, and 204 (20.1%) patients aged ≥50. A total of 547 (53.89%) patients are male and 468 (46.11%) patients are female. Among these patients, 753 (74.14%) were white, 164 (16.16%) were black, and 98 (9.66%) were others. As for tumor size, 142 (13.99) patients were less than 5cm, 427 (42.07%) patients were between 5 and 10cm, and others were more than 10cm. In the primary site, 768 (75.67%) patients were located in the limbs, 130 (12.81%) patients were located in the axial, and 117 (11.53%) patients were located in other parts. In the entire cohort, the pathology of 128 (12.61%) patients was high grade and of 887 (87.39%) patients was low grade. The T stage was divided into T1 (41.28%), T2 (56.16%), and T3 (2.56%). The treatment methods taken by patients are different. A total of 920 (90.64%) patients received surgery, while others did not.

**Table 1:** Demographics and clinical characteristics of the whole training and validation cohorts

Variables	Type	Total (*n*=1015)	Validation (*n*=405)	Train (*n*=610)	*P* value
Age	<20	484 (47.68%)	201 (49.63%)	283 (46.39%)	0.535
	20–49	327 (32.22%)	123 (30.37%)	204 (33.44%)	
	≥50	204 (20.1%)	81 (20%)	123 (20.16%)	
Sex	Male	547 (53.89%)	230 (56.79%)	317 (51.97%)	0.1484
	Female	468 (46.11%)	175 (43.21%)	293 (48.03%)	
Race	White	753 (74.19%)	304 (75.06%)	449 (73.61%)	0.346
	Black	164 (16.16%)	58 (14.32%)	106 (17.38%)	
	Other	98 (9.66%)	43 (10.62%)	55 (9.02%)	
Size	<5	142 (13.99%)	56 (13.83%)	86 (14.1%)	0.514
	5–10	427 (42.07%)	179 (44.2%)	248 (40.66%)	
	≥10	446 (43.94%)	170 (41.98%)	276 (45.25%)	
Primary site	External	768 (75.67%)	320 (79.01%)	448 (73.44%)	0.0695
	Axial	130 (12.81%)	49 (12.1%)	81 (13.28%)	
	Other	117 (11.53%)	36 (8.89%)	81 (13.28%)	
Grade	High	128 (12.61%)	51 (12.59%)	77 (12.62%)	1
	Low	887 (87.39%)	354 (87.41%)	533 (87.38%)	
T	T1	419 (41.28%)	167 (41.23%)	252 (41.31%)	0.8028
	T2	570 (56.16%)	226 (55.8%)	344 (56.39%)	
	T3	26 (2.56%)	12 (2.96%)	14 (2.3%)	
Surgery	No	95 (9.36%)	36 (8.89%)	59 (9.67%)	0.7569
	Yes	920 (90.64%)	369 (91.11%)	551 (90.33%)	

### Kaplan–Meier survival analysis and univariate and multivariate logistic regression analysis

**Fig. 1:**
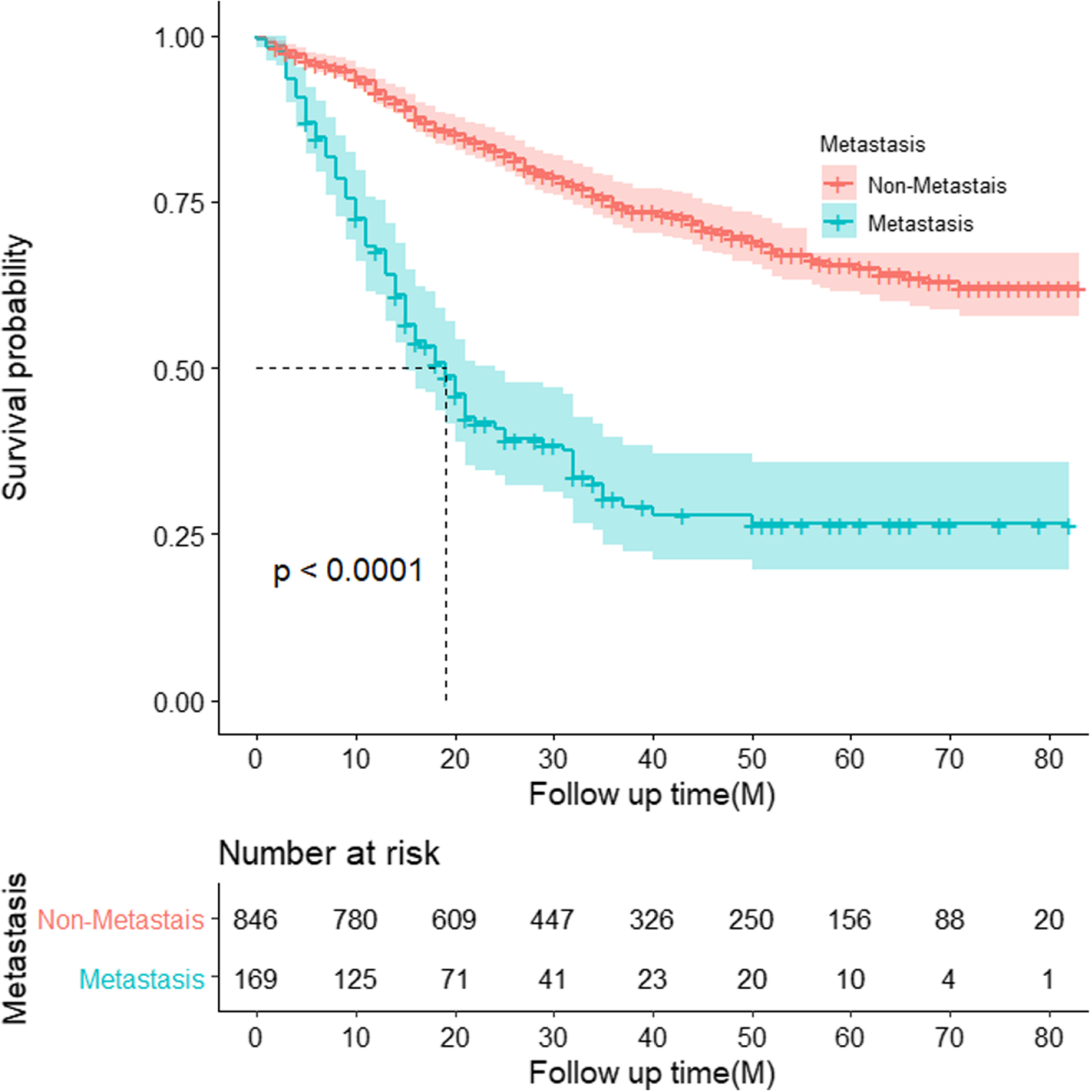
Kaplan–Meier analysis for overall survival in metastasis and non-metastasis osteosarcoma

The results of Kaplan–Meier survival analysis show that in the overall survival of osteosarcoma patients, patients with metastasis are significantly poor than that of patients without metastases (Fig. 1). Logistic regression analysis was applied to filter factors which affect metastasis. According to the univariate logistic regression analysis and the log-rank test of osteosarcoma patients (Table 2), external, low grade, and high T stage have more metastasis risk. However, patients who received surgery and aged 20–49 have less risk to metastasis. There were no significant differences in race and sex.

**Table 2:** Univariate and multivariate logistic regression analysis in the training cohort

Variables		Univariate analysis		Multivariate analysis	
		OR (95%CI)	*P* value	OR (95%CI)	*P* value
Age			0.001		0.027
	<20	Reference		Reference	
	20–49	0.38 (0.21, 0.66)	<0.001	0.45 (0.24, 0.84)	0.012
	≥50	0.62 (0.35, 1.12)	0.114	0.58 (0.27, 1.26)	0.169
Sex			0.547		
	Male	Reference			
	Female	0.87 (0.56, 1.36)	0.547		
Race			0.902		
	White	Reference			
	Black	1.03 (0.58, 1.84)	0.909		
	Other	0.66 (0.27, 1.61)	0.363		
Primary site			<0.001		0.019
	External	Reference		Reference	
	Axial	0.81 (0.42, 1.57)	0.527	0.44 (0.18, 1.05)	0.065
	Other	0.12 (0.03, 0.49)	0.003	0.2 (0.04, 0.94)	0.041
Size			<0.001		
	<5	Reference			
	5–10	3.15 (1.08, 9.16)	0.035		
	≥10	5.22 (1.83, 14.84)	0.002		
Grade			<0.001		0.032
	High	Reference		Reference	
	Low	7.72 (1.86, 32.01)	0.005	3.8 (0.88, 16.3)	0.073
T			<0.001		0.027
	T1	Reference		Reference	
	T2	2.81 (1.65, 4.76)	<0.001	1.92 (1.09, 3.4)	0.024
	T3	8.7 (2.75, 27.55)	<0.001	3.72 (1.03, 13.47)	0.045
Surgery			<0.001		<0.001
	No	Reference		Reference	
	Yes	0.19 (0.11, 0.34)	<0.001	0.12 (0.05, 0.25)	<0.001

On the basis of univariate logistic regression analysis, factors with *P*<0.05 that may affect the metastasis risk of osteosarcoma were selected to perform multivariate logistic regression analysis to identify independent risk variables. Finally, age, size, primary site, grade, T stage, and surgery were used to perform multivariate logistic regression analysis. The multivariate analysis demonstrated that age 20–49 (OR 0.45; 95%CI (0.24,0.84), *p* = 0.012), axial (OR 0.44; 95%CI (0.18,1.05); *p* = 0.065), primary site except external and axial (OR 0.2; 95%CI (0.04,0.94); *p* = 0.041), and surgery (OR 0.12; 95%CI (0.05,0.25); *p* < 0.001) were independent protect variables and low grade (OR 3.8; 95%CI (0.88,16.3); *p* =0.032) and T3 (OR 3.72; 95%CI (1.03,13.47); *p* =0.045) were independent risk factors.

### Construction and validation of nomogram

**Fig. 2:**
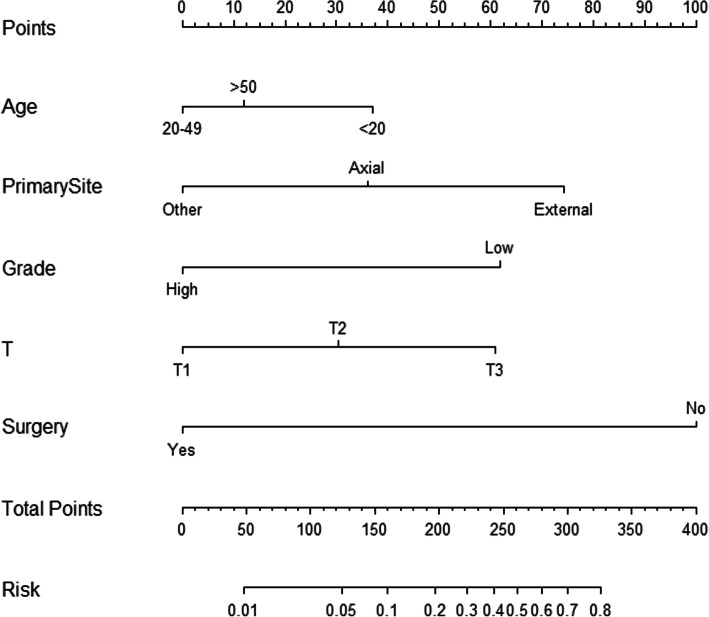
Nomogram to predict metastasis in osteosarcoma

Based on the results of multivariate logistic regression analysis, we construct the nomogram with age, primary site, T stage, grade, and surgery (Fig. 2).

**Fig. 3:**
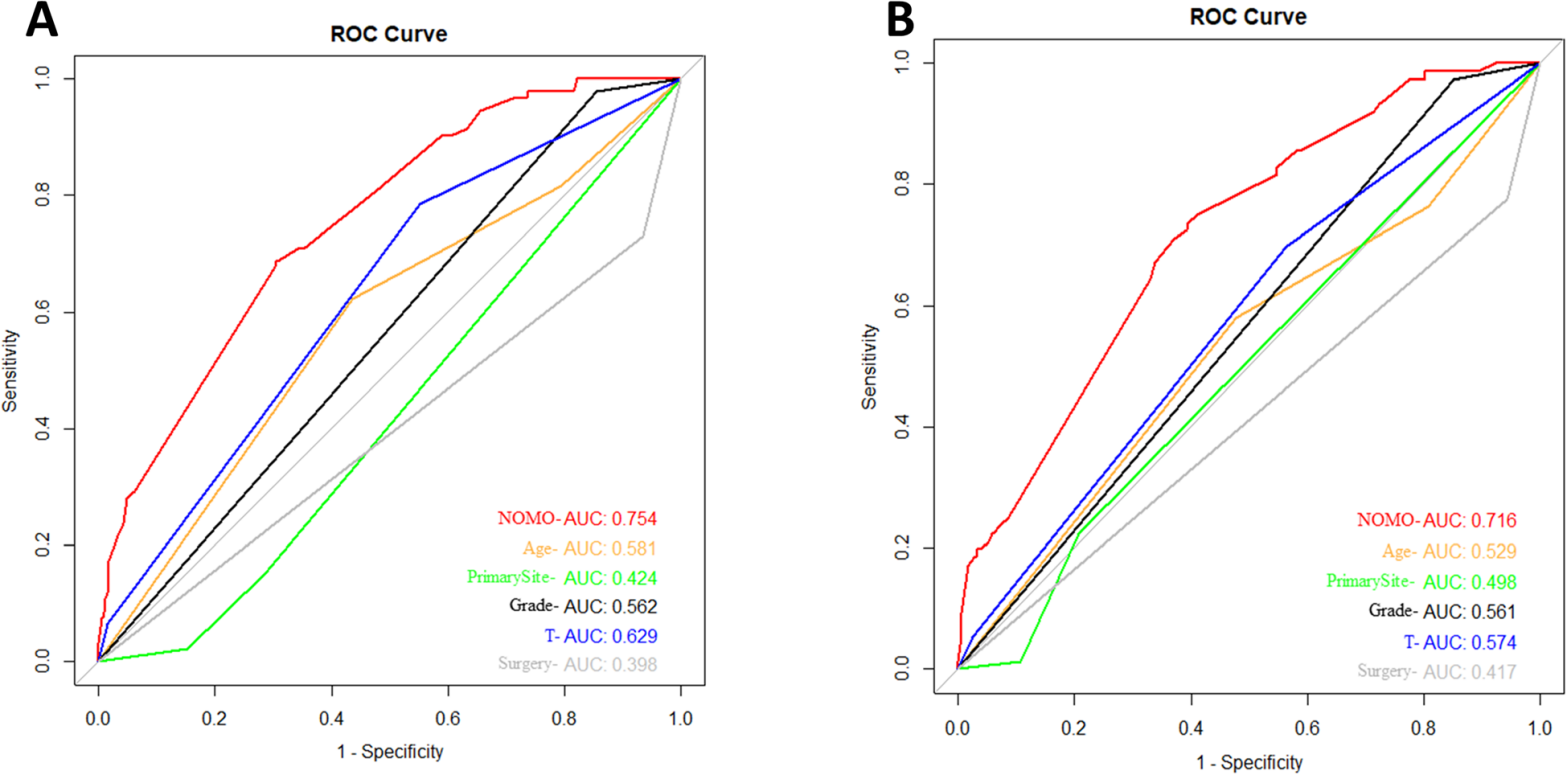
Validation of the nomogram with ROC curve. **a** The training cohort. **b** The validation cohort

**Fig. 4:**
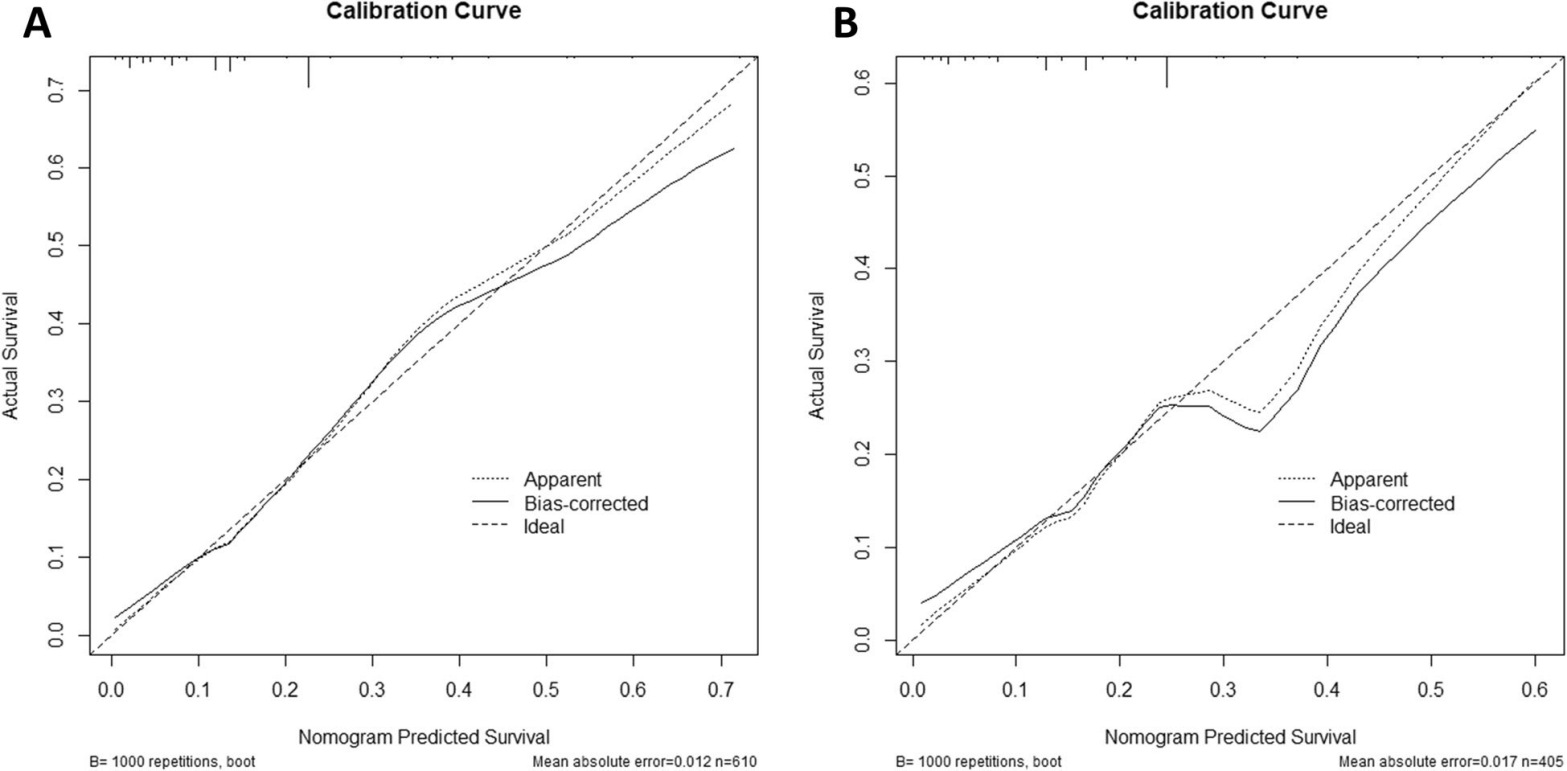
Calibration plot of the nomogram for the probability of metastasis. **a** The training cohort. **b** The validation cohort

In order to verify the accuracy of the nomogram, we performed internal and external validation through concordance indices (C-index) and calibration plots. The C-indexes of training and validation cohort are 0.754 and 0.7169 (Fig. 3). It means that the prediction of the nomogram is great for osteosarcoma metastasis. Besides, the prediction and observed outcomes for tumor metastasis which the calibration plots show in Fig. 4 are highly consistent both in the training and validation cohort. These results indicate that nomogram shows significantly superior prediction performance.

## Discussion

In 2020, there will be approximately 3600 new bone tumor patients, and about 1720 patients will die from the malignant cancer in America [13]. Osteosarcoma is the most common cancer type. Former studies focused on finding factors that influence the osteosarcoma prognosis to evaluate the overall survival or cancer-specific survival [14, 15]. There is still no study which focuses on the metastasis in osteosarcoma, which is the most important factor for cancer prognosis [16]. However, nomogram has been applied to predict metastasis in other cancer types. Study by Cai et al. combined age, grade, histology, T stage, lymph node metastasis, and tumor size to predict the metastasis in T1 and T2 gallbladder cancer [17]. In pancreatic ductal adenocarcinoma, demographic and clinicopathological characteristics were used to construct nomogram to evaluate metastasis [18]. However, there is still no similar study in osteosarcoma.

The nomogram is an accurate and convenient mathematical model which can predict a specific end point [19]. It is a reliable tool to quantify and assess risks, which can help clinicians better diagnose and determine treatment options. Therefore, it is imperative to evaluate metastasis status through nomogram. Study by Cao et al. [20] has identified several metastasis-associated genes and this way may be effective for OS patients. The skeletal microenvironment composed of mesenchymal stem cells (MSC), osteoblasts, osteoclasts, osteocytes, fibroblasts, fat cells, etc., provides an ideal growth place for many cancers. For example, the most common metastasis sites for breast and prostate cancer are bones [21, 22]. This special tumor microenvironment is an ideal place for the occurrence, development, and metastasis of osteosarcoma. The tumor microenvironment also changes with age, tumor location, size, and grade. However, genomic sequencing is quite expensive and not every patient can afford it. Therefore, it is urgent to develop an economical model to evaluate metastasis status.

In this study, age, tumor size, primary site, grade, T stage, and surgery were meaningful factors for OS metastasis in univariate logistic regression analysis. After stepwise logistic regression, age, grade, primary site, T stage, and surgery were identified as most meaningful factors. Age is usually thought to be a factor which affects prognosis [23, 24]. Nowadays, it has been proved to be related to lung metastasis in OS [3]. Our results also display that OS patients aged 20–49 have fewer metastases comparing to children and older patients. We think that may be triggered by body development status. The child’s body is not fully developed and old people are aging. Human aging is accompanied by cell aging, which includes changes of nuclear genome instability, protein, and metabolism [25, 26]. These changes may be involved in the occurrence and development of tumors [27]. Tumor grade is the description of a tumor based on how abnormal the tumor cells and the tumor tissue look under a microscope. It is an indicator of how quickly a tumor is likely to grow and spread according to National Cancer Institute (https://www.cancer.gov/about-cancer/diagnosis-staging/prognosis/tumor-grade-fact-sheet). In our study, the low grade can be a risk factor for metastasis. Except tumor grade, there are other potential candidates initially associated with the tumor for identifying high-risk patients, such as tumor size, location, histological subtype, and biological characteristics [28]. The study by Kim et al. [28] demonstrated that initial tumor size is related to the histological response and survival time of patients with osteosarcoma. Surgery is the core treatment for osteosarcoma [29]. Although surgery effect affecting by many factors, complete resection of the primary tumor blocks the progression of tumors including metastases in some extent [30, 31]. In our study, results demonstrate that surgery effectively prevents OS metastasis. However, the ability of a single factor to affect the metastasis of osteosarcoma is limited, so we combine multiple prognostic factors to predict metastasis. The nomogram which can combine the multiple variables to predict tumor risk has long been widely accepted.

Finally, we developed the nomogram to predict metastasis with age, primary site, T stage, and surgery. According to the set ratio, each prognostic factor has a corresponding value. Based on the personalized information and its corresponding value, we can get a total score, which is used to predict metastasis risk. For example, for patients with osteosarcoma, you can find the corresponding points in the nomogram based on patients’ information, add all the points, and correlate the total score with the probability of the event we are trying to predict.

Our research also has some limitations. We only searched the patient’s medical records in the SEER database. Although the SEER database represents 30% of the US population, it is inevitable that some patients have missing information; if we include other databases, some grey literature resources, meeting records, or non-English articles, we may find some other information that can make prediction results more accurate, in spite of the possibility is very small. Second, some patients with osteosarcoma lack some information to analyze, for example, surgical margin status, and the radiotherapy and chemotherapy data in the SEER database are limited, which may lead to inaccurate inferences.

In conclusion, the nomogram is more accurate when tested in internal and external validation cohorts. If others can use our nomogram in some prospective studies or other databases, it may be more conducive to verify the accuracy of this model. The nomogram developed by us helps clinicians better predict metastasis risk and determine postoperative treatment strategies for patients with osteosarcoma.

## Data Availability

The raw data are from Surveillance, Epidemiology, and End Results (SEER) database.

## Abbreviations

OS: Osteosarcoma
SEER: Surveillance, Epidemiology, and End Results
C-index: Concordance index
ROC: Receiver operating characteristic
